# Supplementary material for: Evaluating Large Language Models for Translating Multimodal Phenotype Documentations into Executable EHR Phenotyping Algorithms
Source: medRxiv. 2026 May 22:2026.05.20.26353690. Preprint. [Version 1] doi: 10.64898/2026.05.20.26353690 (PMC13228752; doi:10.64898/2026.05.20.26353690)
Supplement: 1 [file NIHPP2026.05.20.26353690V1-supplement-1.pdf]

medRxiv preprint doi: https://doi.org/10.64898/2026.05.20.26353690; this version posted May 22, 2026. The copyright holder for this preprint (which was not certified by peer review) is the author/funder, who has granted medRxiv a license to display the preprint in perpetuity.
It is made available under a CC-BY 4.0 International license .

Supplementary Information

Evaluating Large Language Models for Translating Multimodal Phenotype Documentations into Executable EHR Phenotyping Algorithms

Chao Yan[1,*], Yi Xin[2,*], Wu-Chen Su[1], Srushti Gangireddy[1], Shravani Durbhakula[3], Stephen P. Bruehl[3], Alyson L. Dickson[4], Lang Li[5], QiPing Feng[4], Bradley A. Malin[1,2,6,7], Tyler Derr[2], Wei-Qi Wei[1,2,†]

[1]Department of Biomedical Informatics, Vanderbilt University Medical Center, Nashville, TN, USA
[2]Department of Computer Science, Vanderbilt University, Nashville, TN, USA
[3]Department of Anesthesiology, Vanderbilt University Medical Center, Nashville, TN, USA
[4]Department of Medicine, Vanderbilt University Medical Center, Nashville, TN, USA
[5]Department of Biomedical Informatics, The Ohio State University, Columbus, OH, USA
[6]Department of Biostatistics, Vanderbilt University Medical Center, Nashville, TN, USA
[7]Department of Electrical and Computer Engineering, Vanderbilt University, Nashville, TN, USA

* Co-first authors
† Corresponding author; correspondence should be addressed to wei-qi.wei@vumc.org.

medRxiv preprint doi: https://doi.org/10.64898/2026.05.20.26353690; this version posted May 22, 2026. The copyright holder for this preprint (which was not certified by peer review) is the author/funder, who has granted medRxiv a license to display the preprint in perpetuity.
It is made available under a CC-BY 4.0 International license .

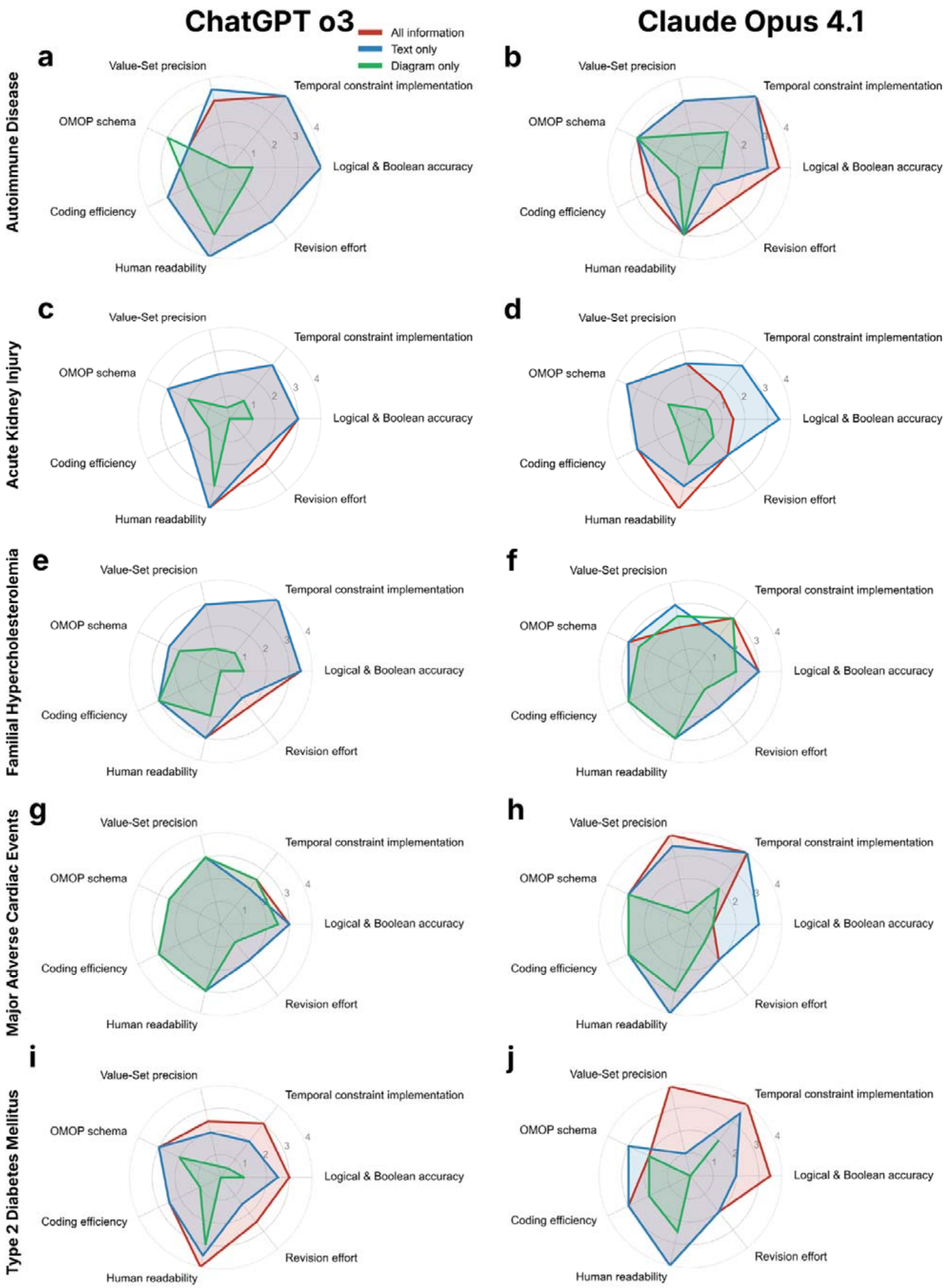

Supplementary Fig. 1: Expert evaluation of LLM-generated OMOP SQL queries. Mean ratings for seven dimensions stratified by two LLMs and five clinical phenotypes: **ab,** Autoimmune disease. **cd,** Acute kidney injury. **ef,** Familial hypercholesterolemia. **gh,** Major adverse cardiac events. **ij,** T2DM: type 2 diabetes mellitus.

medRxiv preprint doi: https://doi.org/10.64898/2026.05.20.26353690; this version posted May 22, 2026. The copyright holder for this preprint (which was not certified by peer review) is the author/funder, who has granted medRxiv a license to display the preprint in perpetuity.
It is made available under a CC-BY 4.0 International license .

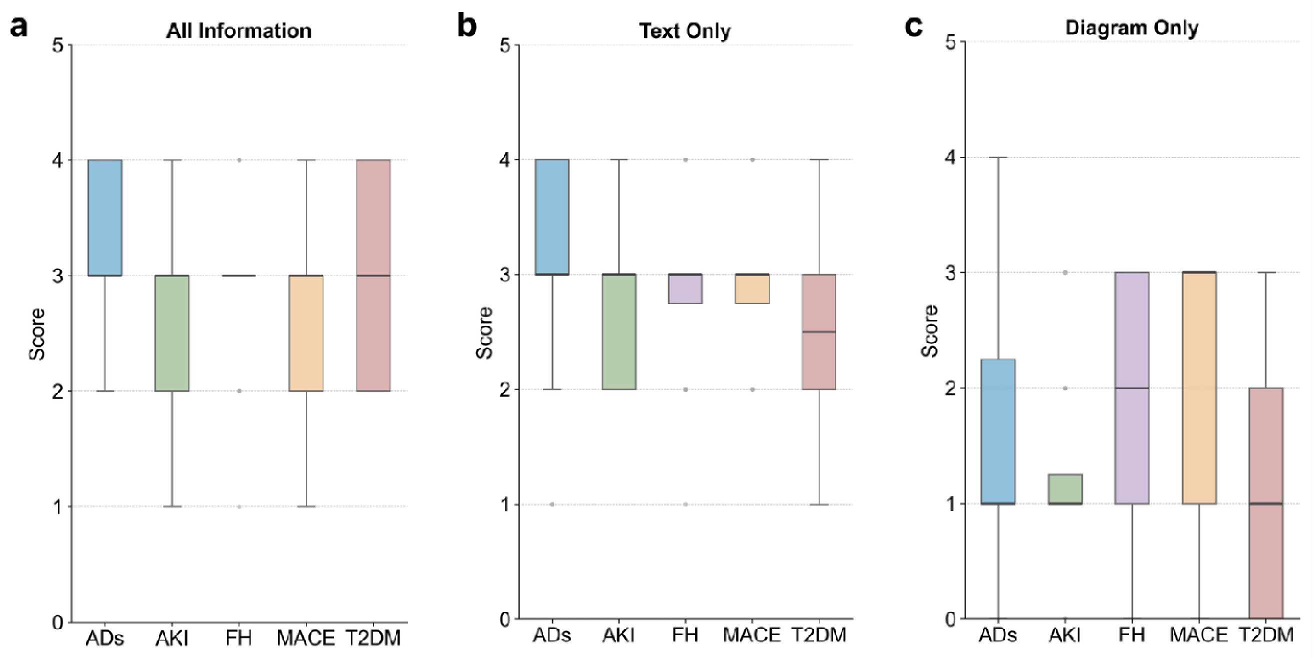

Supplementary Fig. 2: Expert evaluation of LLM-generated OMOP SQL queries across OpenAI o3 and Claude Opus 4.1 for five clinical phenotypes stratified by information settings: a, All information; **b,** Text only; **c,** Diagram only. ADs: autoimmune disease; AKI: acute kidney injury; FH: familial hypercholesterolemia; MACE: major adverse cardiac events; T2DM: type 2 diabetes mellitus.

Supplementary Table 1: Prompt templates for stepwise LLM-based phenotype implementation. A sequential four-prompt chain decomposes the task into two phases: algorithm extraction (prompts 1&2), in which clinical logic is parsed from PDF documentation, and SQL generation (prompts 3&4), in which the extracted logic is rendered as executable OMOP-conformant queries.

Prompt 1:

You are an expert in disease phenotyping. Your task is to extract the phenotyping algorithm for identifying cases and controls for {disease_name} from the attached PDF file.

Let's think step by step:

1. List all the considered criteria. Do not refer to tables, figures, or other sources.
2. Extract how these criteria are combined.
3. Derive the algorithm.
4. Ensure optimal readability by using informative comments and organized headers.

Instructions to follow:

1. Use “AND”, “OR”, “NOT” and other necessary logical operators to organize the algorithm clearly and precisely.
2. Use Observational Medical Outcomes Partnership (OMOP) concepts for diagnoses, symptoms, procedures, laboratory tests, medications, etc.
3. When both names and codes for considered concepts are provided, only use codes and do not use names.
4. Use SQL style to organize the final algorithm.
5. Do not use ellipsis in the algorithm.
6. Do not refer to tables, figures, or other sources.
7. Make sure descriptive comments and headers are added to enhance human readability.

Prompt 2:

[illegible]

medRxiv preprint doi: https://doi.org/10.64898/2026.05.20.26353690; this version posted May 22, 2026. The copyright holder for this preprint (which was not certified by peer review) is the author/funder, who has granted medRxiv a license to display the preprint in perpetuity.
It is made available under a CC-BY 4.0 International license .

version.

Instructions to follow:

1. Use “AND”, “OR”, “NOT” and other necessary logical operators to organize the algorithm clearly and precisely.
2. Use Observational Medical Outcomes Partnership (OMOP) concepts for diagnoses, symptoms, procedures, laboratory tests, medications, etc.
3. When both names and codes for considered concepts are provided, only use codes and do not use names.
4. Use SQL style to organize the final algorithm.
5. Do not use ellipsis in the algorithm.
6. Do not refer to tables, figures, or other sources.
7. Make sure descriptive comments and headers are added to enhance human readability.

Prompt 3:

Now you need to generate the executable SQL query of the algorithms above so that cases and controls of the phenotype can be accurately found from an EHR database that follows the OMOP Common Data Model. Do not generate tables. Make sure descriptive comments and headers are added to enhance human readability.

Prompt 4:

Verify that the generated SQL queries and all their details are grounded in the algorithms extracted above. Make sure these SQL queries are executable in EHR databases that follow the OMOP Common Data Model. If not, correct all errors and generate the final queries without asking further questions. Do not generate tables. Make sure descriptive comments and headers are added to enhance human readability.

Supplementary Table 2: **Evaluation criteria used by clinical experts.**

How to use the rubric

1. **Prepare:** Read the PDF file of each PheKB phenotype, marking key concepts, IDs, logic, temporal rules, and thresholds.
2. **Score:** For each dimension, score the integer 0-4 that best matches the performance of the LLM-generated SQL queries.
3. **Document:** Note all errors/issues directly when providing the score. This will be used to perform error analysis and will be summarized into our paper.

1. Logical & Boolean Accuracy

What to assess: Compare AND/OR/NOT nesting, inclusion/exclusion blocks, and joins against the PDF’s algorithm narrative. Confirm there is no logical inversion or omission.

Score	Interpretation for this dimension
0	Logical structure contradicts the PDF
1	Major nesting or block errors that alter cohort definition
2	Minor bracket/alias mistakes affecting edge cases
3	Logic matches PDF; only cosmetic tweaks needed
4	Logic matches exactly and is organized clearly

2. Temporal Constraint Implementation

What to assess: Check that every time window, sequence requirement, and date arithmetic (e.g., “two diagnoses within 365 days”) is implemented with correct boundaries and units.

Score	Interpretation for this dimension
0	Temporal rules missing, reversed, or implement a hallucinated window that is not in the PDF
1	Temporal logic present but window length or units wrong by >30 % **or** direction (before/after) incorrect
2	Window lengths/units and sequence order correct, but edge inclusivity/exclusivity mishandled or unclear
3	Windows, sequencing, and edge conditions correct; only minor clarity or parameter-tuning issues remain

medRxiv preprint doi: https://doi.org/10.64898/2026.05.20.26353690; this version posted May 22, 2026. The copyright holder for this preprint (which was not certified by peer review) is the author/funder, who has granted medRxiv a license to display the preprint in perpetuity.
It is made available under a CC-BY 4.0 International license .

4	All windows, sequencing, and edge conditions correct; parameters are clearly surfaced and well documented

3. Value-Set Precision (Numeric & Categorical Thresholds)

What to assess: Verify that numeric cut-offs, categorical filters, and unit casts match the PDF exactly, including boundary operators (≥ vs >) and null handling. This does not include temporal constraints in 4.

Score	Interpretation for this dimension
0	Thresholds missing, flipped, or hallucinated **or** units clearly wrong
1	Threshold present but off by >10 % **or** unit/data type mismatch **or** null handling absent
2	Threshold value and unit correct, but casting, boundary operator, or null logic ambiguous
3	Threshold, unit, casting, boundary operator, and null logic correct; minor documentation tweaks only
4	Threshold, units, casting, boundary operator, and null logic all correct and unambiguous. Ready to run with no edits

4. OMOP Schema & Domain Correctness

What to assess: Determine whether the SQL references the proper OMOP tables and domains for each clinical concept (e.g., condition_occurrence for diagnoses, drug_exposure for medications, measurement for labs). Confirm correct joining keys (person_id, visit_occurrence_id) and appropriate use of standard vocabulary tables. Hallucinated or mismatched tables/domains count against accuracy.

Score	Interpretation for this dimension
0	>30 % of concept references point to incorrect, non-standard, or hallucinated OMOP tables/domains **or** join keys are broadly mis-applied, blocking execution
1	15 – 30 % of references incorrect **or** multiple hallucinated tables/domains; some joins use wrong keys
2	5 – 14 % of references incorrect **or** a single join key mis-used; vocabulary tables inconsistently applied
3	All concepts mapped to correct tables/domains, joins use proper keys; minor portability concerns (e.g., hard-coded schema qualifiers)
4	All concepts mapped to correct OMOP tables/domains; joins use correct keys; no site-specific assumptions or missing vocabulary joins

5. Coding Efficiency

What to assess: Look for unnecessary Cartesian joins, full-table scans, or other anti-patterns that would hamper performance on large datasets.

Score	Interpretation for this dimension
0	Unbounded joins or runaway scans likely to fail or time out on any sizable dataset
1	Multiple avoidable full-table scans or missing join predicates that will severely slow execution
2	Mostly efficient but with one prominent anti-pattern or missing index causing moderate slowdown
3	Sensible joins, predicates, and selective filters; minor optimizations still possible; expected to run acceptably on 1 – 10 M-row datasets
4	Perfect in efficiency and no optimization is necessary

6. Human Readability

What to assess: Evaluate indentation, alias clarity, section comments, and presence of a high-level header describing purpose and inputs/outputs.

Score	Interpretation for this dimension
0	Unformatted SQL; no comments; aliases cryptic
1	Basic indentation but still difficult to scan; few or no comments

medRxiv preprint doi: https://doi.org/10.64898/2026.05.20.26353690; this version posted May 22, 2026. The copyright holder for this preprint (which was not certified by peer review) is the author/funder, who has granted medRxiv a license to display the preprint in perpetuity.
It is made available under a CC-BY 4.0 International license .

2	Consistent indentation and clear aliases; sporadic comments provide limited guidance
3	Well-commented blocks that map code sections to algorithm steps; layout easy to follow
4	Readable layout with concise header summarizing purpose, inputs, and outputs

7. Revision Effort for Local Deployment (Readiness)

What to assess: Estimate how much time a typical analyst must spend editing the query to run it on certain OMOP instance (e.g., schema qualifiers, vocabulary database names).

Score	Interpretation for this dimension
0	>3 hours or structural rewrite required
1	1 – 3 hours of edits
2	30 – 60 min; mostly schema qualifiers
3	10 – 30 min; minor tweaks
4	≤10 min; essentially plug-and-play

Supplementary Table 3: **Interrater agreement between two clinical experts, measured by Cohen's kappa for five diseases**. ADs: autoimmune disease; AKI: acute kidney injury; FH: familial hypercholesterolemia; MACE: major adverse cardiac events; T2DM: type 2 diabetes mellitus.

LLM	Phenotyping algorithm	Cohen's kappa (quadratic)	LLM	Phenotyping algorithm	Cohen's kappa (quadratic)
OpenAI o3	ADs	0.9275	Claude Opus 4.1	ADs	0.9356
	AKI	0.8094		AKI	0.8027
	FH	0.8222		FH	0.6316
	MACE	0.5714		MACE	0.9605
	T2DM	0.6364		T2DM	0.9569
	**Overall**	0.7974		**Overall**	0.8977
